# New Insights into Muscle Function during Pivot Feeding in Seahorses

**DOI:** 10.1371/journal.pone.0109068

**Published:** 2014-10-01

**Authors:** Sam Van Wassenbergh, Billy Dries, Anthony Herrel

**Affiliations:** 1 Department of Biology, Universiteit Antwerpen, Antwerpen, Belgium; 2 Department of Biology, Ghent University, Gent, Belgium; 3 Department of Veterinary Medical Imaging and Small Animal Orthopaedics, Ghent University, Merelbeke, Belgium; 4 Département d’Ecologie et de Gestion de la Biodiversité, Centre National de la Recherche Scientifique/Muséum National d’Histoire Naturelle, Paris, France; University of Münster, Germany

## Abstract

Seahorses, pipefish and their syngnathiform relatives are considered unique amongst fishes in using elastic recoil of post-cranial tendons to pivot the head extremely quickly towards small crustacean prey. It is known that pipefish activate the epaxial muscles for a considerable time before striking, at which rotations of the head and the hyoid are temporarily prevented to allow energy storage in the epaxial tendons. Here, we studied the motor control of this system in seahorses using electromyographic recordings of the epaxial muscles and the sternohyoideus-hypaxial muscles with simultaneous high-speed video recordings of prey capture. In addition we present the results from a stimulation experiment including the muscle hypothesised to be responsible for the locking and triggering of pivot feeding in seahorses (m. adductor arcus palatini). Our data confirmed that the epaxial pre-activation pattern observed previously for pipefish also occurs in seahorses. Similar to the epaxials, the sternohyoideus-hypaxial muscle complex shows prolonged anticipatory activity. Although a considerable variation in displacements of the mouth via head rotation could be observed, it could not be demonstrated that seahorses have control over strike distance. In addition, we could not identify the source of the kinematic variability in the activation patterns of the associated muscles. Finally, the stimulation experiment supported the previously hypothesized role of the m. adductor arcus palatini as the trigger in this elastic recoil system. Our results show that pre-stressing of both the head elevators and the hyoid retractors is taking place. As pre-activation of the main muscles involved in pivot feeding has now been demonstrated for both seahorses and pipefish, this is probably a generalized trait of Syngnathidae.

## Introduction

Constraints on muscular power generation have led to the evolution of mechanisms relying on elastic recoil to perform movements that are fast and powerful enough to capture exceptionally elusive prey. Examples of elastic energy storage and release during prey capture can be found in tongue-projection mechanisms in chameleons [1]–[3], salamanders [4] and toads [5], hyoid motions during intra-oral prey processing in knifefish [6], raptorial appendage strikes in mantis shrimps [7], [8]), carnivorous terrestrial plants employing trap-snapping [9] or tentacle-snapping [10], and aquatic carnivorous plants generating a sudden trap inflation to produce suction [11]. Since these movements rely on the same mechanical principles as catapults (i.e., a slow increase in strain in an elastic material during preparation, followed by a triggered release of the stored elastic energy during the launch), they are sometimes referred to as biological catapults [12].

Syngnathid fishes (pipefishes, seahorses, and seadragons) use movements driven by catapult-like mechanics to rapidly capture elusive prey such as small crustaceans [13]–[16]. This action involves a very fast dorsal rotation of the head that brings the mouth close to the prey [13], [17]–[19]. Subsequently, suction is produced to draw the prey into the mouth cavity [18]. This dual-phase mechanism (i.e., head rotation followed by suction) is called pivot feeding [17]. Syngnathids can generate very high angular accelerations of the head because this motion is powered by the recoil of the epaxial tendons [14]. The latter study showed that the epaxial muscle of the pipefish *Syngnathus leptorhynchus* is active for at least 200 milliseconds prior to the start of head rotation, allowing the storage of energy by stretching these tendons. Afterwards, the head is suddenly rotated over more than 20 degrees in less than 5 milliseconds, leaving prey almost no chance to react.

**Figure 1:**
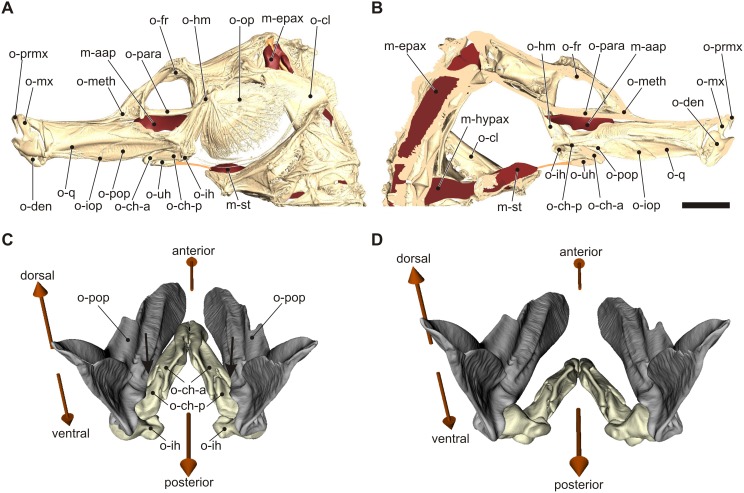
Anatomy of the head and illustration of the hyoid locking hypothesis in *Hippocampus reidi*. Left lateral view of the CT-reconstruction of the cranium with addition of the stimulated muscles based on histological sections (A), a medial view of the left side of the head with the opercular bone removed (B). Below, a detailed view on the hyoid and suspensorium illustrate the morphological configuration hypothesised to be responsible for locking of hyoid and head rotation where the posteriolateral part of the anterior ceratohyals lies dorsally of one of the medial grooves of the adducted preopercular bones (C), and the unlocked configuration where the ceratohyals are free to rotate ventrally after slight abduction of the preopercula (D). Orange lines in (A) and (B) represent tendinous connections. The arrows indicate the most likely contact region between the ceratohyals and the preopercula. Abbreviations: m-aap, adductor arcus palatini; m-epax, epaxial muscle; m-hypax, hypaxial muscle; m-st, sternohyoideus muscle; o-ch-a, anterior ceratohyal bone; o-ch-p, posterior ceratohyal bone; o-cl, cleithrum; o-den, dentary bone; o-fr, frontal bone; o-hm; hyomandibular bone; o-ih, interhyal bone; o-iop, interopercular bone; o-meth, mesethmoid bone; o-mx, maxillary bone; o-op, opercular bone; o-para, parasphenoid bone; o-pop, preopercular bone; o-prmx, premaxillary bone; o-q, quadrate bone; o-uh, urohyal bone. Scale bar, 5 mm.

Despite the recent advances in our understanding of the feeding mechanics of syngnathid fishes, several hypotheses related to motor control remain untested. Firstly, the timing of activation of the epaxial musculature in syngnathids has only been studied in a single species of pipefish. Still, as considerable variation exists within the Syngnathidae in cranial and post-cranial morphology [20] as well as in the kinematics of head rotation [19], data from sister taxa of this pipefish species are needed to confirm whether the same pattern of epaxial muscle activity is widespread within Syngnathidae. Secondly, while muscle activation patterns have only been measured for the epaxial muscle [14], a similarly long tendon is present on the ventral side of the head: the tendon of the sternohyoideus-hypaxial muscle [14], [16] which inserts on the hypohyals and ceratohyals of the hyoid via the urohyal (Figure 1A, B). Consequently, we hypothesised that an activation pattern comparable to that measured for the epaxial muscles may also occur in the sternohyoideus-hypaxials. In addition, two aspects of the prey-capture kinematics suggest a pre-activated state: (1) we argued that the absence of dorsal bending of the trunk during the loading phase suggests that the stress in the epaxial region is countered by forces at the ventral (i.e. hypaxial) side of the vertebral column [14], and (2) the initial angular acceleration of the hyoid is extremely fast compared to other fish [18].

Previous analyses of kinematics showed a considerable amount of variation in the magnitude of head rotation within the seahorse *Hippocampus reidi* [18], [21]. We therefore hypothesised that seahorses are capable of active control over the distance they rotate their head as a function of prey distance. If so, this should be reflected in the recruitment and/or activation characteristics of the epaxials (head rotation accelerators) and/or the sternohyoideus-hypaxials (which theoretically can act as head decelerators in the final phase of cranial rotation; [16], [17]). Consequently, to increase the magnitude of head rotation, we predict an increased level of epaxial muscle activation and/or a decreased level of sternohyoideus-hypaxial activation.

Finally, the current hypothesis on the triggering mechanism of pivot feeding in syngnathids suggests that the head is prevented from rotating dorsally as long as the hyoid is prevented from rotating ventrally in between the preopercular bones [16], [17] (Figure 1C, D). The coupled motion of the hyoid and neurocranium results from a four-bar linkage involving these two elements [13], [17], [21], [22]. Since the preopercula are adducted by the adductor arcus palatini muscle (Figure 1A, B), activation offset in this muscle coupled with activity onset of its antagonist, the levator arcus palatini, may trigger release of the hyoid and neurocranium for rotation. So far, however, no experimental data have been collected to evaluate this hypothesis.

The goal of this study was to test the following hypotheses by means of simultaneous recording of electromyography and high-speed video in the seahorse *Hippocampus kuda*: (1) epaxial pre-activation is not restricted to pipefish; (2) sustained pre-activation is also present in the sternohyoideus-hypaxial muscle complex; (3) seahorses display control of mouth displacement distance in relation to prey distance, and if so (4) this motor control is reflected in the electromyographic patterns of the epaxials and/or the sternohyoideus-hypaxials. Finally, we evaluate the proposed mechanism of the relaxation of preopercular adduction as the trigger of the pivot feeding catapult system via a muscle stimulation experiment in *Hippocampus reidi*.

## Materials and Methods

### Ethics statement

All experiments were approved by the animal ethics committee at the University of Antwerp (Ethische Commissie Dierproeven, Permit Number: 2011-69, approved 11/2011), under the condition of strictly minimizing the number of individuals used in the experiments.

### Study animals

Seahorses of the species *Hippocampus kuda* Bleeker, 1852 (electromyography analysis; 2 individuals) and *Hippocampus reidi* Ginsburg, 1933 (electric stimulation experiment; 2 individuals plus 1 individual for a fatigue test) were used. These species were chosen based on availability in captive-bred stocks of local commercial aquarium trade. The animals were housed in a large aquarium (200l) with a constant temperature (24°C), salinity (35 ppt) and photoperiod (12∶12) and were fed daily with freely suspended, defrosted *Neomysis vulgaris*. The *H. kuda* individuals had head lengths ( = snout tip to back of the coronet) of 14.1 mm (individual 1) and 15.2 mm (individual 2); head lengths of the *H. reidi* individuals were measured 31.9 mm, 32.4 mm and 34.8 mm.

### Electromyography (EMG) and high-speed video recordings

**Figure 2:**
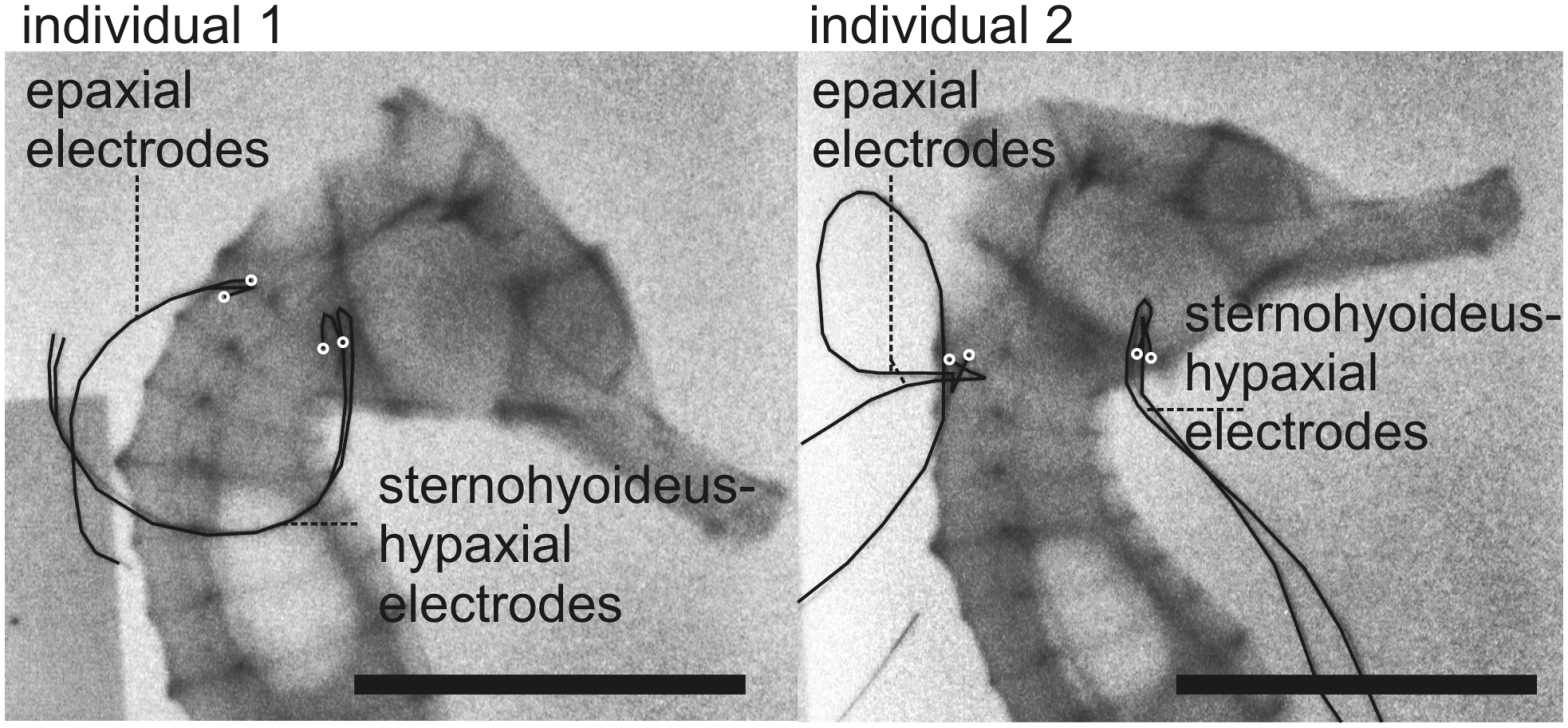
X-ray images showing the placement of the electrodes for both individuals in the electromyography analysis. The uncoated electrode tips are indicated by white circles. Scale bars, 10 mm.

**Figure 3:**
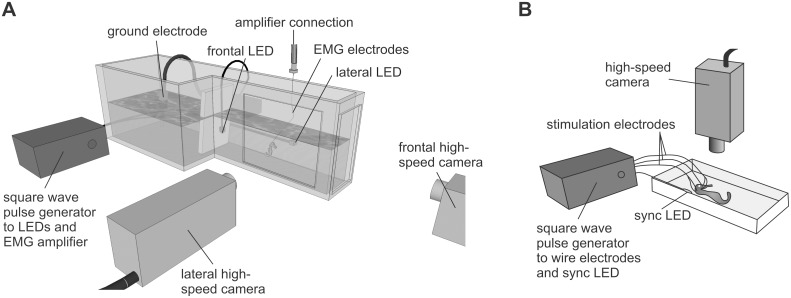
Experimental set-up for electromyography (A) and for the stimulation experiment (B).

The seahorses were anaesthetized with tricaine methanesulfonate (MS222) prior to insertion of bipolar stainless steel (Advent Research Materials Ltd, Halesworth, England) twisted-hook, quadruple Teflon-insulated electrodes with cross-sectional diameters of 75 µm via hypodermic needles. The approximately 0.5 mm long uncoated tips of the electrodes were inserted into two muscles: the epaxial muscle at the level of the second anterior bony ring of the trunk, and mid-belly in the sternohyoideus-hypaxial muscle (Figure 2). Electrode positions were verified by lateral view radiographs (Ajex 9020H X-ray generator, Ajex Meditech, Seoul, Korea; EVA digital sensor, Imageworks, New York, USA) overlaid on graphical reconstructions of the anatomy (Figure 1A, B). Since no other muscles are present in the close proximity of the insertion positions, correct electrode placement could be safely confirmed. These radiographs also showed that the final pole spacing varied between 0.4 to 0.8 mm (Figure 2). Next, the animal was transferred to a small aquarium (30l), which contained a narrow section to restrict the movement during the recording session (Figure 3A). The electrode signals were amplified by a factor of 10 using Gould Universal preamplifiers (Gould Electronics, Eichstetten, Germany; filter bandpass 1 to 10 000 Hz) and Honeywell Accudata 117DC amplifiers (Honeywell International Inc., Morristown, USA) before being recorded digitally on tape using a TEAC 145 T DAT recorder (TEAC Corporation, Tokyo, Japan). EMG data was monitored and exported to ASCII files using TEAC QuickVu software, and further analyzed in Microsoft Excel. All EMGs were recorded within 36 hours from the start of the anaesthesia.

Dual view high-speed videos were recorded simultaneously with EMG: a Redlake MotionPro HR1000 camera (IDT, Tallahassee, USA) filmed in lateral view at 1000 frames per second, and a Redlake Motionscope M3 camera filmed in frontal view at 500 frames per second (Figure 3A). The camera views were orthogonal and perpendicular to the aquarium walls, which minimizes the distortion of the imaging volume. To allow synchronization between the EMG and the videos, LEDs in view of each camera were powered by a Grass S48 squared wave pulse generator (Grass Technologies, West Warwick, USA), of which the output was connected to an amplifier channel that was recorded on the DAT. Three infrared 140-LED (Scene Electronics, Shenzhen, China) arrays provided the necessary illumination.

**Figure 4:**
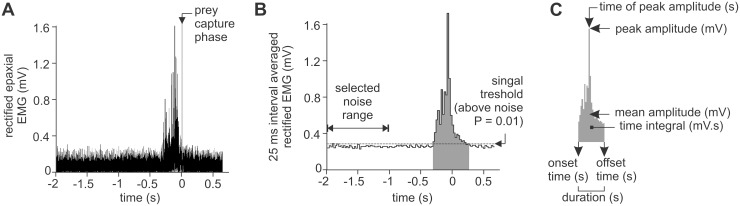
Calculation steps in the analysis of the recorded EMGs. The rectified EMG signal with the duration of the prey capture phase (narrow grey bar) is shown in (A). In (B) noise range selection is illustrated, with determination of the onset and offset times for 25 ms intervals exceeding the signal threshold of the 99% confidence limit of the noise. In (C), the six EMG variables are shown.

Time zero was set as the time of the video image in which the first feeding motion, depression of the hyoid, was visible. Since the signal-to-noise ratios of the EMGs were relatively low due to the small size of the muscles, the following calculation steps had to be performed to determine the onset and offset times of the activity bursts of the muscles. After rectification (Figure 4A), a period of about 1 s only containing noise and occurring well before the strike (>1 s) was selected. Signal threshold was set at the 99% confidence limit (mean +2.576 standard deviations) of the voltage amplitude of this noise period. Next, the EMG amplitudes were averaged for 25 ms intervals. This interval length was selected as shorter intervals regularly yielded multiple crossings of the signal threshold within one burst of EMG. Falsely identified signal or noise intervals were corrected if they were neighbored by two intervals of an opposite result. The onset time then corresponded to the start of the first 25 ms interval value exceeding this noise voltage treshold (Figure 4B). The offset time was the end of the latest 25 ms interval exceeding this treshold (Figure 4B). For the onset-to-offset interval, the following variables were calculated (Figure 4C): duration, mean amplitude, peak amplitude, time of peak amplitude, and time integral. The latter variable was included since the time integral of EMG bursts has shown to correlate particularly well with muscle force during feeding movements [23], [24]. EMGs were analysed from 12 successful feeding sequences of individual 1, and 13 successful feeding sequences of individual 2.

### Video analysis

**Figure 5:**
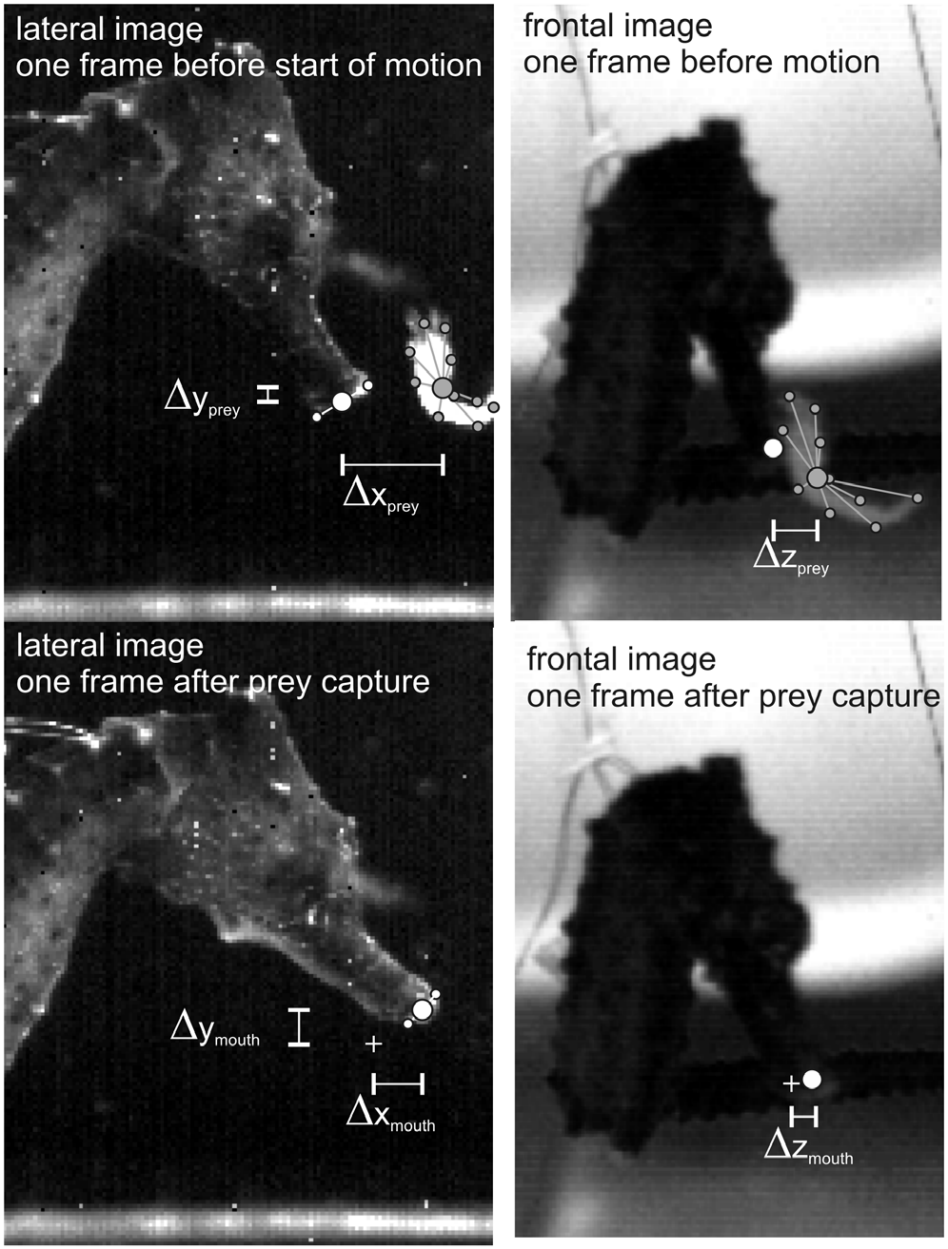
Example of the calculation of prey distance and mouth travel distance. The formulae 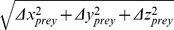 and 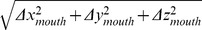 were used to calculate these two respective variables via digitization of landmarks at the mouth and at the contours of the prey on video images shot with two perpendicular cameras. In the lower panels, “+” indicates the position of the centre of the mouth at one frame before the start of head rotation. See text for further information.

To study whether motor patterns are related to prey distance in *Hippocampus kuda*, two variables were quantified based on the dual view high-speed video images: prey distance and mouth travel distance. The three-dimensional prey distances were calculated from video stills at a single frame before the start of the movement (Figure 5). This distance runs from the centre of the mouth (position in between a dorsal and ventral landmark at the mouth in the lateral images; Figure 5) to the centre of the prey (centroid of ten approximately equally spaced landmarks along the contours of both the lateral and frontal view video images). The three-dimensional mouth travel distances were calculated between video stills at one frame before the start of the feeding motion and at one frame after the capture of the prey (Figure 5), using the coordinates of the mouth centre. Two-dimensional landmark coordinates were obtained using Didge (version 2.2.0, A. Cullum, Creighton University, USA) for the lateral view (*xy*-axes) and frontal view (*yz*-axes) to determine 3D coordinates (*xyz*). As the scaling factor increases with the distance of object away from the camera lens, this distance was measured from the other camera view. Next, scale factors were calculated for the front and the back of the camera view, and linear interpolations using the relative position of the mouth and prey in between front and back view planes yielded the scaling factor used in the further analyses. Prey distances could be determined for 9 (individual 1) and 12 (individual 2) prey captures. Mouth travel distances could be determined for 11 (individual 1) and 12 (individual 2) prey captures. All variables (i.e. EMG, prey distance, mouth travel distance) could be gathered for 9 (individual 1) and 10 (individual 2) prey captures.

### Stimulation experiment

Three *Hippocampus reidi* specimens were sacrificed using an overdose of MS222 just prior to each stimulation experiment. Nickel-chrome thin wire bipolar electrodes (diameter 50 µm) were implanted in the epaxial, sternohyoideus-hypaxial and adductor arcus palatini muscles (Figure 1A, B) on each side (a total of six electrodes). Single trains of electric pulses were given with a Grass S48 stimulator [25]. Train duration was 0.3 s (corresponding to the minimal burst durations measured in the current study), pulse frequency and duration were respectively 330 Hz and 0.15 ms, and the stimulation voltage was 5 V. Because we wanted to prevent damage to the muscles, we choose a relatively low stimulation voltage. Consequently, it is likely that not all motor unit pools of the muscles under study were maximally activated, resulting in a decreased performance relative to *in vivo* observations. Muscle fatigue was avoided by waiting at least 5 minutes between the stimulation events.

**Figure 6:**
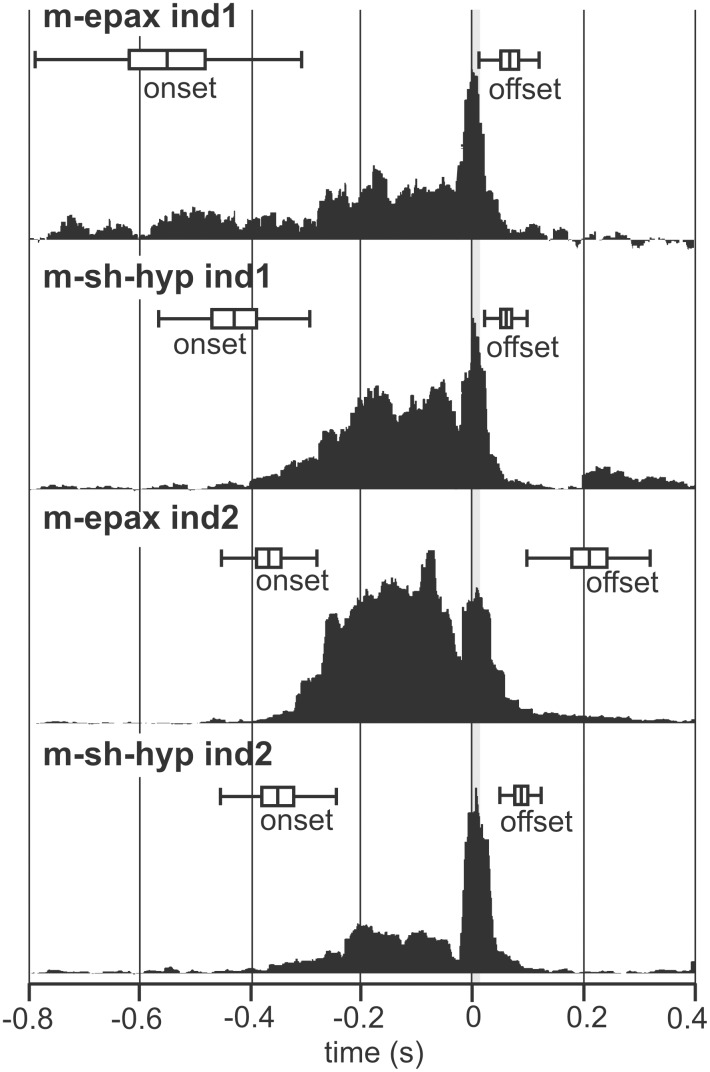
Activation patterns for the epaxial muscle (m-epax) and the sternohyoideus-hypaxial muscle (m-st-hyp). The start of the prey-capture movement equals time = 0 s with a grey bar indicating the duration of the head rotation phase. Boxes (mean±s.e.) and whiskers (mean±s.d) denote the onset and offset times. Black profiles represent the within-individual means of the integrated rectified EMGs above or below the noise level. Mean profile amplitudes are separately scaled to improve clarity.

During the stimulation experiments the seahorse was placed in a dissection tray with sea water and positioned on its side, with the trunk touching the bottom of the tray but the head free to move. A Redlake Motionscope M3 camera was used to record the movements of the head in lateral view together with an on- and offset indicator of the stimulator (Figure 6B). Five stimulation events were recorded in two individuals (10 events in total). Next, the electrodes leading to the left and right adductor arcus palatini muscle were cut and an additional five stimulation events for each animal were recorded.

Two video landmarks were digitized frame by frame using Didge: 1) the tip of the snout and 2) transition of the mesethmoid and the frontal bone, located just anterior to the eye. With these two variables, the head angle relative to the horizontal axis was calculated. Since the reference frame of this angle is earthbound, the head angle is expressed as this angle minus the initial head angle.

The third animal was used to validate whether head rotation characteristics remained unaffected by the increasing number of stimulation trials. The six muscles of this animal were stimulated for fifteen consecutive trials using the same stimulation parameters and rest times as described above, while movement of the head was recorded. Regressing (ordinary least squares) the maximal head velocity calculated in each trial to trial number gave a slope of 0.12, and no relation between maximal head velocity and the trial number was observed (*R^2^* = 0.002, *P* = 0.87).

### Statistics

To test whether the EMG onset and offset times and durations differ between the two muscles within each individual, two-tailed paired Student T-tests were performed. Unpaired T-tests are used to test whether there are differences between individuals within muscle. To test whether a correlation exists between prey distance and mouth travel distance, and between prey distance or mouth travel distance and the EMG variables, reduced major axis (RMA) models were used (RMA add-in for Excel; University of Ottawa, LPC Freeware). This type of analysis is recommended when the samples of both co-dependent variables include a comparable degree of natural variation, and the *XY* and *YX* line fittings are presumed to display symmetry [26].

Five kinematic variables of the stimulation trials in which the adductor arcus palatini muscles were stimulated, and of the trials in which the adductor arcus palatini muscles were not stimulated, were compared using within each individual using two-tailed T-tests. These variables were (1) the total head rotation, (2) the time between the onset of head rotation and the instant of maximal head rotation, (3) the mean velocity of head rotation, (4) the timing of the instant of maximal velocity, and (5) the time between the first visible movement and the onset of the stimulation.

## Results

### Onset and offset times

The onset times of the epaxial muscle (m-epax) and sternohyoideus-hypaxial muscles (m-st-hyp) did not differ significantly within each individual (ind.1, *P* = 0.16; ind.2, *P* = 0.63) (Figure 6). Activation onset of the epaxial muscle preceded the start of head and hyoid rotation by minimally 0.19 s (average±s.d. = −0.55±0.24 s) and 0.23 s (average±s.d. = −0.36±0.24 s) for individual 1 and 2, respectively. Onset of the hypaxial muscle preceded the first motion by a minimum of 0.30 s (average±s.d. = −0.43±0.14 s) and 0.12 s (average±s.d. = −0.35±0.10 s). Activation lasted until shortly after completion of head rotation (Figure 2). Offset times were significantly greater for the m-epax compared to the m-st-hyp for individual 2 (*P* = 0.005), but this difference was not detected for individual 1 (*P* = 0.77). The second individual showed a later onset and offset time for m-epax only compared to individual 1 (*P* = 0.016, onset; *P* = 0.0005, offset) suggesting individual differences in electrode position within the muscles or control strategies between individuals.

### Kinematic modulation

**Figure 7:**
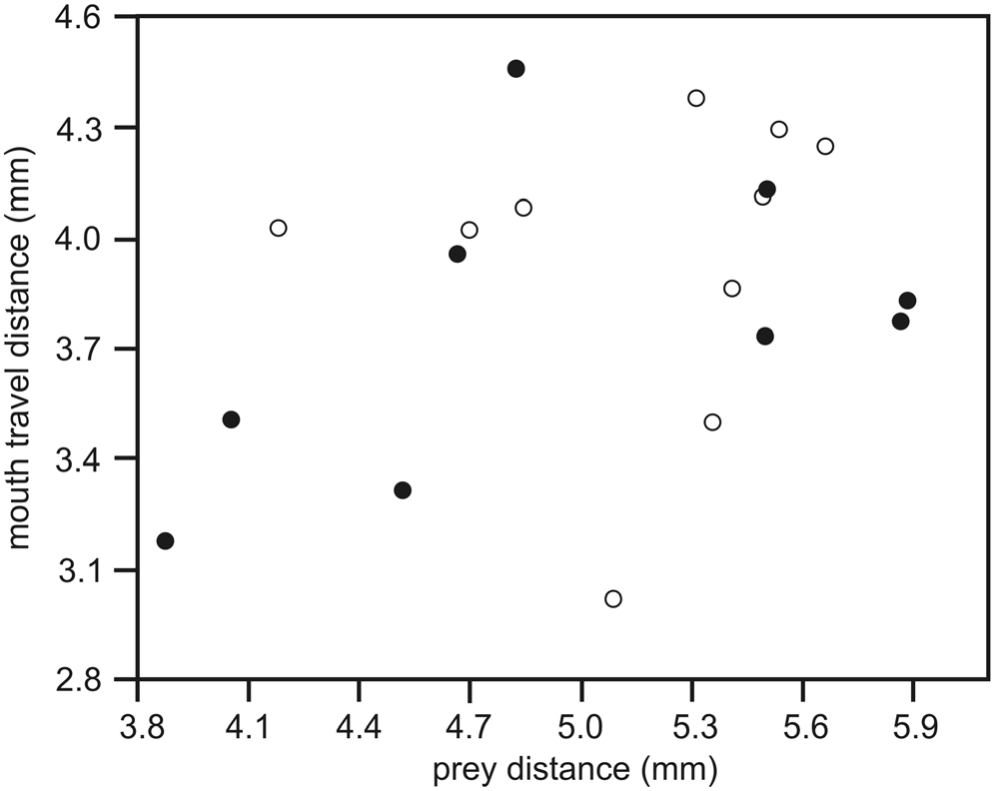
Relationship between prey distance and mouth travel distance. Note that no significant correlations were calculated for each of the individuals (individual 1, filled circles; individual 2, open circles).

Prey distance varied between 3.9 mm and 6.1 mm (average±s.d. = 5.3±0.6 mm), and mouth travel distance ranged from 3.2 mm to 4.7 mm (average±s.d. = 4.0±0.4 mm). No significant correlation was found between prey distance and mouth travel distance when pooling the data from the two individuals (RMA regression slope = 0.67±0.14; *R* = 0.35; *P* = 0.14; data corrected for head length difference between the individuals) (Figure 7). Although both individuals separately showed similar slopes (slope individual 1 = 0.53±0.15, *R* = 0.48; slope individual 2 = 0.90±0.28; *R* = 0.12), these relationships were also not significant (*P* = 0.19 and 0.48 respectively) (Figure 7).

### Motor control as a function of prey distance

No significant correlations were detected between prey distance and any of the variables describing the EMG pattern of the m-epax and m-st-hyp within each individual. The only noteworthy trends were a tendency of later onset and longer time to peak m-epax amplitude with more distant prey in individual 1 (*P* = 0.07 and 0.053, respectively), and a tendency for higher mean amplitudes of m-st-hyp with more distant prey in individual 2 (*P* = 0.053). However, these trends were not found in the other individual.

Variation in mouth travel distance was also not significantly related to the variables describing the EMG pattern of the m-epax and m-st-hyp within each individual. Although the predicted pattern of increased mean EMG amplitude of the m-epax (more powerful dorsal head rotation) combined with a decrease in the mean amplitude of the m-st-hyp (later or less powerful head deceleration) for increased head rotation was indeed observed for both individuals, the correlations were not significant (*P* always>0.12). This predicted pattern could not be observed for the EMG time integral.

### Muscle stimulation

**Figure 8:**
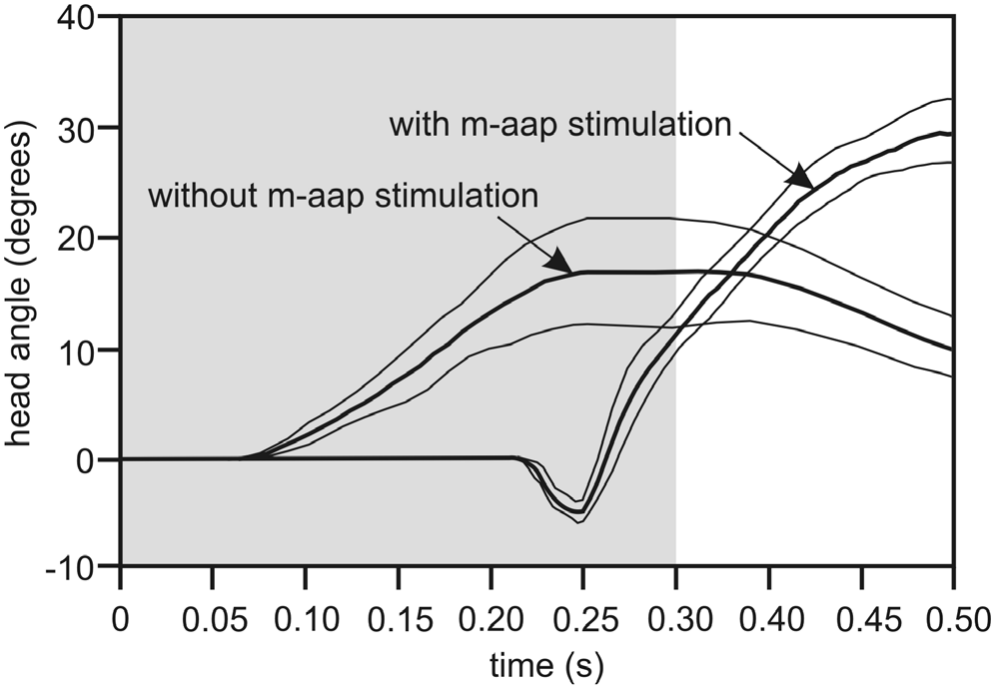
Results of the stimulation experiment. The figure shows the time-dependent kinematic profile of head rotation (mean±s.d.) when the epaxial, hypaxial and adductor arcus palatine muscles are stimulated, and when only the epaxial and hypaxial muscles are stimulated. The grey zone represents the time of stimulation. *N* = 2 individuals, five repetitions per individual and stimulation treatment.

A clear difference was observed in the kinematics of head rotation with and without adductor arcus palatini muscle (m-aap) stimulation in *Hippocampus reidi* (Figure 8). The average maximal head angle observed when the m-aap was stimulated in conjunction with the m-epax and m-st-hyp is 34.1±2.8 deg (mean±s.d.). This angle is significantly larger than the angle of 19.1±1.9 deg, observed when the adductor muscles were not activated (*P*<0.0001 for both individuals). However the average time between the onset of head rotation to maximal head rotation did not differ statistically between both treatments (*P*>0.05 for both individuals).

After the m-aap were activated the resulting average angular head velocity was 584±40 deg s^−1^, which was significantly higher than the angular head velocity of 210±24 deg s^−1^ when the adductor muscles were not stimulated (*P<*0.00001 for both individuals). The average timing at which maximal head velocity was reached was also different in both situations (*P*<0.0001). This timing was 0.012±0.003 ms and 0.12±0.03 ms in the stimulated and non-stimulated situation of the m-aap, respectively.

There was a clear difference between stimulation treatments in timing between the first visible movement and the onset of stimulation (*P<*0.00001 for both individuals) (Figure 8). When the m-aap were stimulated, no movement occurred until an average of 48.90±2.11 ms before the end of stimulation. This means that no movement was observed for approximately 250 ms of stimulation. When the adductor arcus palatini muscles are not stimulated, visible movement occurs after 59±17 ms of stimulation.

## Discussion

Our results indicate that seahorses activate the epaxial muscles a considerable time before (i.e. >0.3 s) the start of the first visible prey capture movement (Figure 6). This finding matches results from pipefish [14], suggesting that this is a generalized syngnathid trait. Epaxial pre-activation allows the animal to strain the left and right epaxial tendons and store elastic energy which can later be used to power the rotation of the head during pivot feeding. The presence of such a power amplification system in seahorses was already suggested by inverse dynamic modelling in juvenile seahorses, which showed an instantaneous peak power of over 3000 W per kg of epaxial muscle required to accelerate the head as observed on high-speed videos [15]. Such high values of instantaneous power output inevitably call for the use of recoil of pre-stressed elastic tissues, as the maximal instantaneous value reported for vertebrate skeletal muscle is 1120 W per kg [27], [28]. The current EMG results confirm the presence of such a mechanism in adults of the species *Hippocampus kuda*.

The hypothesis that the ventral series of muscles inserting on the hyoid (i.e. a complex formed by the sternohyoideus and the hypaxial muscle) would show a sustained pre-activation comparable to that of the epaxial muscles [14] was also confirmed (Figure 6). Since white muscle used for suction feeding in teleost fish (without significant series elastic element) generally need activation times of less than 20 ms to reach half the maximum isometric force upon *in-vitro* tetanus stimulation [29], [30], the observed delay between activation onset and the first hyoid motion of generally over 300 ms (Figure 2) means that considerable retraction force will be exerted on the hyoid prior to the start of the strike at the prey. This inevitably implies some stretching of the sternohyoideus tendon, and elastic recoil and power amplification in the ventral muscle-tendons causing the extremely fast rotation of the hyoid [21] seems likely. Yet, empirical evidence for this can only be given by *in-vivo* strain measurements (e.g. wallabee hopping [31]; frog jumping [12]).

The observed activation patterns of the sternohyoideus-hypaxial muscle rule out the option that this muscle would work as the trigger of the pivot-feeding system of Syngnathidae. This triggering function was proposed by [13] by assuming that the line of action of the sternohyoideus muscle differs from the straight line between the ceratohyal symphysis and the pectoral girdle muscle attachment site formed by the urohyal-sternohyoideus compex. A closer morphological inspection of this system in the seahorse *Hippocampus reidi* [21] and the pipefish *Syngnathus acus* [17] failed to reveal such a system: like in other teleosts, the urohyal typically ossifies within the tendon of the sternohyoideus and will therefore be positioned in the working line of sternohyoideus muscle-tendon. The EMG pattern observed here, confirms that the force from the sternohyoideus does not result in a subtle configuration change in the hyoid-neurocranium four-bar linkage causing the “quick-release” of the system. In addition, no correlations between EMG timing and amplitude variables with the motion onset were found.

The presented kinematic data could not prove that a feed-forward control mechanism is involved in *H. kuda* to fine-tune the amount of head rotation to the distance of the prey. Similar to previous studies on other syngnathid fishes [18], [19], we have shown that there is a considerable strike-to-strike variation in the magnitude of head rotation, but it could not be confirmed for *H. kuda* that this variation is related to the distance from the mouth to the prey at the onset of the strike (Figure 7). Yet, as suction is only effective to draw prey into the mouth from a relatively short distance in stationary predators (e.g. [32]), an accurate positioning of the mouth close to the prey is critical to improve prey capture success. Alternatively, being able to modulate head rotation magnitude would reduce the time and energy spent in pursuing the prey by broadening the range head positions with respect to the prey from which successful strikes can be initiated. Motor control to adjust the degree of head rotation during suction feeding has been shown previously for catfish [33]. The reason why we assume that this would require a feed-forward mechanism in sygnathids, rather than feed-back control as described for other fishes [34], [35], is the extremely short time (generally <5 ms) between the onset of the movement and the actual ingestion of the prey. This time is shorter than the range of reaction latencies measured for fish, which vary from 5 to 40 ms among different species as illustrated for escape responses by Eaton and Hackett [36]. However, our data were not conclusive on the existence of such a feed-forward mechanism during pivot-feeding in *H. cuda*.

In addition, no indication of an active control of the epaxial and sternohyoideus-hypaxial muscles in function of prey distance or mouth travel distance was detected in our EMG data. In other words, it remains unclear how syngnathids manage to vary their head rotation amplitude. We hypothesised that either an increased activation of the epaxials for farther-reaching strikes would occur, or that that tension would build faster in the ventral series of muscles, tendons and ligaments by altered activation patterns of the sternohyoideus-hypaxial muscles. Neither of these hypotheses could be confirmed with our data. However, the signal-to-noise ratio of our measurements may have been too low due to the relatively small size of the muscles (Figure 4A). This may have introduced error in the electromyographic variables preventing us from detecting correlations between muscle activation patterns and the relatively small kinematic changes. Alternatively, control may be manifested in other regions of these muscles (only a single electrode per muscle was used), or in other muscles (e.g., protractor hyoidei, adductor arcus palatini).

The results from the stimulation experiment are in line with the current hypothesis [17] that the main muscle responsible for locking and triggering the release of the hyoid is the adductor arcus palatini muscle (m-aap). Activation of the m-aap blocks the hyoid from moving ventrally for a considerable time, which upon release increased the acceleration, peak velocity and head rotation magnitude (Figure 8). Although the precise *in-vivo* behaviour could not be reproduced in this artificial stimulation experiment, it clearly points to an important role of suspensorium abduction in the locking and triggering process. Electromyographic data of the m-aap during prey capture would be extremely informative in this respect, but the small size of this muscle and its position medial of the suspensorium make this measurement untractable. Previous work has shown that the mass of the m-aap is considerably higher than needed if its role during feeding would be limited to reposition the abducted suspensorium by compressing the buccal cavity to its resting state after feeding [37]. Combined with this observation, our stimulation experiments support the role of the m-aap as the lock and trigger of the elastic recoil mechanism during pivot feeding in seahorses.

In conclusion, the hypothesis that fast head rotation during pivot feeding is driven by catapult-like mechanics of the musculoskeletal system [13] was confirmed for seahorses. The pre-activated state of the head-rotator muscles for generally more than 0.3 s prior to the onset of the strike allows storage of energy in the epaxial tendons (Figure 6). As this is similar to previous results on pipefish [14], this suggests that this type of feeding mechanics is probably widespread among syngnathid species. The present study is the first to show that a pre-activated state is also present in the hyoid-retractor muscles at the ventral side of the head (Figure 6). As long as the rotation of the hyoid is prevented by suspensorium adduction (Figure 1C, D) while experiencing retraction force, the dorsal and ventral pre-activated muscles will work antagonistically, the head remains static, and elastic elements in series with these muscles can be strained. The adductor arcus palatini muscles are probably of crucial importance in locking and release triggering of this mechanism, as shown by our stimulation experiment (Figure 8). After the release of the hyoid, quick dorsal rotation of the head will bring the mouth close to the prey. The present study could not demonstrate that prey located farther away from the mouth elucidated a higher magnitude of head rotation (Figure 7), and neither did our data show altered muscle activity patterns that could be causing the observed kinematic variation.
